# Invasive Candidiasis in Non-Hematological Patients

**DOI:** 10.4084/MJHID.2011.007

**Published:** 2011-01-25

**Authors:** Malgorzata Mikulska, Matteo Bassetti, Sandra Ratto, Claudio Viscoli

**Affiliations:** Division of Infectious Diseases, San Martino University Hospital, Genoa, Italy

## Abstract

*Candida* is one of the most frequent pathogens isolated in bloodstream infections, and is associated with significant morbidity and mortality. In addition to haematological patients, there are several other populations with a substantial risk of developing invasive candidiasis (IC). These include patients undergoing prolonged hospitalisation with the use of broad-spectrum antibiotics, those fitted with intravascular catheters, admitted to both adult and neonate intensive care units (ICU) or gastrointestinal surgery wards and subjects with solid tumours undergoing cytotoxic chemotherapy. As a general rule, every immunocompromised patient might be at risk of *Candida* infection, including, for example, diabetic patients.

The epidemiology of species responsible for IC has been changing, both at local and worldwide level, shifting from *C. albicans* to non-albicans species, that can be intrinsically resistant to fluconazole (*C. krusei* and, to some extent, *C. glabrata*), difficult to eradicate because of biofilm production (*C. parapsilosis*) or than might acquire resistance to azole during therapy.

Delaying the specific therapy has been shown to increase morbidity and mortality, but traditional microbiological diagnosis is poorly sensitive and slow. Thus, culture-based treatment may result in therapy started too late. In order to reduce the mortality in IC, several management strategies have been developed: prophylaxis, empirical and pre-emptive therapy. Compared to prophylaxis, the latter approaches allow to reduce the use of antifungals by targeting only patients at very high risk of IC. Non-invasive serological markers and scores based on clinical prediction rules such as the presence of risk factors or *Candida* colonisation, have been developed with the aim of allowing prompt initiation of treatment. Although the use of these diagnostic tools in pre-emptive strategies is promising, the performance and cost-effectiveness should be tested in large trials.

Agents recommended for initial treatment of candidemia in severely ill patients include echinocandins and lipid formulations of amphotericin B, while stable patients without risk factors for azole-resistance might be treated with fluconazole.

## Introduction

*Candida* is a yeast responsible for the majority of fungal infections in humans. This fungus causes pathologies of different severity, ranging from mucocutaneous infections to invasive disease that can involve any organ. The incidence of invasive candidiasis (IC), particularly candidemia, has increased significantly in recent years and *Candida* spp. is now the fourth most common pathogen isolated in blood cultures in the US.^1^ In Europe it ranks among the ten most frequently isolated pathogens.^2^,^3^ Candidemia is a life-threatening infection with high morbidity and mortality.^4^–^7^ Even in the most recent studies, crude mortality rates reached 50–60% in critically ill patients,^8^–^10^ although attributable mortality can be substantially lower.

Immunocompromised patients, such as those affected by solid tumours or haematological malignancies are at high risk for developing *Candida* infection. However, the widespread use of fluconazole prophylaxis in haematological and stem cell transplant settings might be responsible for a decreased incidence of invasive *Candida* infections in these populations.^11^ On the contrary, patients with multiple severe comorbidities, undergoing gastrointestinal surgery or admitted to ICU constitute now the largest population at risk for developing candidemia.^12^ In fact, IC can affect up to about 10% of all critically ill subjects.^13^,^14^ Fungal infections are being increasingly diagnosed in these patients, because advances in medical science now allow patients in desperate underlying conditions to survive. However, this is not obtained without a price, such as the development of infectious complications. Therefore, the population of subjects vulnerable to a range of infections is increasing and this trend will likely continue.

From a clinical point of view, *Candida* causes bloodstream infections, sometimes with endophtalmitis, followed by peritonitis and other abdominal infection and endocarditis. A matter of debate can be how often a blood culture positive for *Candida* represents the external sign of a deep-seated infection, or it is simply a bloodstream infection without localisation. Most of the patients included in studies on epidemiology or treatment of invasive candidiasis had candidemia (approximately 68–90%), with or without localisation, while peritonitis was the second most common disease (approximately 7–30% of subjects).^9^,^15^,^16^ In a recent French study, isolated candidemia, IC with candidemia and IC without candidemia accounted each for 1/3 of all episodes of IC.^9^ Additionally, *Candid*a accounted for approximately 3% of all surgery-related peritoneal infections, both community-acquired and nosocomial.^9^

On the of main points regarding invasive *Candida* infection is the fact that delaying antifungal treatment significantly increases mortality.^17^–^20^ Even 12–24 hours delay can result in twofold increase in crude mortality rate in candidemia.^21^,^22^ However, nosocomial fungal infections have one of the highest rates of inappropriate therapy, that consists mostly of omission of including an antifungal in the initial empirical therapy and use the of inadequate doses, all of which have been associated with increased mortality.^12^,^21^–^23^ Additionally, the estimated cost of each episode of IC in hospitalised adults is tremendous.^24^,^25^ Thus, high awareness of this infection, early diagnosis and appropriate prompt therapy remain the cornerstone of treatment.

During the last decade several new antifungal drugs have been developed and obtained approval for treatment of *Candida* infections. Therefore, treating a candidemia has become a difficult exercise, because of the need to make the appropriate choice at the appropriate time. In the following lines we will try to discuss epidemiology, risk factors, diagnosis and management of IC in non-haematological patients.

## Epidemiology of invasive candidiasis

The epidemiology of *Candida* infections, both on a worldwide scale, and more importantly on the local level, has significant implications for the management of these infections.

During the past two decades, most hospitals have reported a progressive shift in the species of *Candida*. In the past, almost all the isolates responsible for bloodstream infections were *C. albicans*, whereas in recent years a growing proportion of episodes of candidemia have been caused by *Candida* species other than albicans.^26^–^31^ Although, *C. albicans* remains the predominant strain in most countries,^9^,^32^,^33^ non-albicans species are increasingly common and in some adult ICUs they were responsible for over 50% of candidemias.^29^,^34^ The most common non-albicans species are *C. parapsilosis* and *C. glabrata*, followed by *C. tropicalis* and *C. krusei*.^9^,^29^,^35^–^37^ Rare species reported to cause candidemia include *C. lusitaniae*, *C. guilliermondii,* and *C. rugosa*.^12^,^35^

Numerous studies have tried to find reasons for this shift and several risk factors have been associated with the emergence of non-albicans species.^30^,^38^,^39^ It is likely that the widespread use of fluconazole can predispose patients to the development of infections due to species that are intrinsically resistant to azoles or have developed resistance during treatment. Indeed, the previous use of fluconazole has been found to be a risk factor for the presence of non-albicans fungemia in many studies,^29^,^30^,^40^ even though others did not find the same association.^28^ In particular, risk factors for candidemia due to *C. parapsilosis* include the presence of in-dwelling devices, hyperalimentation and neonatal age.^35^ The specific risk factors associated with IC and with different *Candida* species are outlined in table 1.

The overall rise in the incidence of non-albicans strains is alarming, since there are important differences among species. Specifically, the main difference between *C. albicans* and *C. krusei* or *C. glabrata* is the resistance to the most frequently used antifungal, i.e. fluconazole.^41^ Therefore, species identification and the knowledge of local epidemiology of *Candida* strains causing candidemia is of utmost importance for guiding appropriate empirical therapy. In vitro susceptibility testing of clinical isolates of *Candida* might prove valuable for guiding therapy in patients who have received prior antifungal treatment or who are not responding to first line therapy, especially if performed by experience microbiologists.

## Risk factors for invasive candidiasis and predictive scores

The predominant source of invasive *Candida* infections is endogenous, from superficial mucosal and cutaneous colonisation to haematogenous dissemination,^42^ although cases of exogenous transmission due to contaminated materials or transmission from healthcare workers to patients and from patients to patients have been described.^43^–^46^ The suppression of the normal bacterial flora of the gastrointestinal tract by broad spectrum antibiotic therapy allows the yeast to proliferate and long-term and high density colonisation has been shown to predispose to candidemia.^47^,^48^ Numerous other conditions, frequent in hospitalised patients, such as steroid treatment and poor control of blood glucose concentrations (diabetes) have been described. In addition, parenteral nutrition, intravascular catheters or ischemia and reperfusion, may damage the integrity of the skin or gastrointestinal mucosa, with traslocation and bloodstream invasion. In particular, as much as one third of patients with recurrent gastrointestinal perforations, anastomotic leaks or necrotising pancreatitis develop IC (table 1).^49^,^50^

The effort to identify patients who are at high risk of developing IC has been made in order to reduce mortality by offering them prophylaxis, empirical or pre-emptive treatment. Once risk factors have been reported, they were combined to create reliable risk prediction scores.

Candida colonisation index (CI), reported in 1994, was studied in a surgical population with the aim of predicting patients who would develop IC,^48^ and was used as a base for pre-emptive therapy.^51^ Although it is highly predictive for IC, its routine use has been limited by workload required and consequent costs.

In 2006, Leon and colleagues described their Candida Score (CS) system, that was helpful to select patients who could benefit from early antifungal therapy (those with CS > 2.5 were almost 8 times more likely to develop IC than those with CS < 2.5).^52^ Subsequently, the same group validated their CS in a prospective multicenter trial that included 1107 patients admitted for at least 7 days to ICU.^53^ CS was calculated as follows: 1 point for the presence of parenteral nutrition, surgery or multifocal *Candida* colonization, 2 points for severe sepsis. In patients with Candida Score <3, the incidence of IC was 2.3%, thus allowing to withhold empirical antifungal treatment. On the contrary, one of four patients with a CS of 5 developed IC.^53^

Another clinical risk prediction score was developed by Ostrosky-Zeichner and colleagues. In this study, systemic antibiotic treatment or central venous catheter, combined with two or more of additional five parameters (parenteral nutrition, dialysis, major surgery, pancreatitis, treatment with steroids or other immunosuppressive agents), were able to identify patients with candidemia, with positive and negative predictive values of 10% and 97%, respectively.^54^

Finally, Dupont and colleagues studied a predictive score for peritoneal *Candida* infection in an ICU population and found that the presence of 3 out of 4 factors (female gender, upper gastrointestinal tract origin of peritonitis, intraoperative cardiovascular failure and previous antibiotic therapy) had positive and negative predictive values of 67% and 72%, respectively.^55^

## Diagnosis of candidemia

Blood cultures remain the mainstay for the diagnosis of candidemia, although sensitivity is not optimal and the time from the blood sample collection to the microbiological response of a growing yeast is long. Furthermore, at least 24–48 hours are required for species identification and susceptibility testing. Traditional cultures from sterile sites other than the bloodstream (e.g. peritoneum), remain useful for the diagnosis IC, but more sensitive and more rapid diagnostic methods are needed.

In recent years, non-invasive markers have been investigated, which include serological markers (mannan, antimannan and (1,3)-beta-D-glucan) and polymerase chain reaction. Although the mannan and antimannan commercially available ELISA tests have been marketed for almost 10 years, the only data derive from a single-centre studies that differ significantly in terms of sensitivity and specificity.^56^–^59^ The (1,3)-beta-D-glucan test has been marketed more recently in Europe and in the US. Despite promising results in various cohorts, no large prospective study able to evaluate sensitivity, specificity, and especially cost-effectiveness, has been performed.^60^,^61^ The main problems of the routine use of (1,3)-beta-D-glucan are its high cost and high rate of false positive results. Indeed, (1,3)-beta-D-glucan is ubiquitous in nature contamination can be caused by concomitant bacterial bloodstream infections, presence of surgical gauzes, use of glucan-containing membranes for haemofiltration and use of albumin or immunoglobulins.^62^ For example, in a study that focused on the validation of the Candida Score, (1,3)-beta-D-glucan testing was performed in a subgroup of 240 patients with *Candida* species colonisation or invasive fungal infection.^53^ For a cut-off of 75 pg/ml, good sensitivity of 77.8% was reported, but the specificity was low (52.7%). In particular, among patients with a positive result, only 12% developed documented invasive candidiasis. However, a positive (1,3)-beta-D-glucan result is one of microbiological criteria defining a probable invasive fungal infection according to 2008 definitions of invasive fungal disease published by the European Organization for Research and Treatment of Cancer and the Infectious Diseases Mycoses Study Group (EORTC/MSG).^63^

Finally, two new rapid methods are available for species identification and they include matrix-assisted laser desorption ionization time-of-flight mass spectrometry (MALDI-TOF MS) and fluorescence in-situ hybridization (FISH).

## Management of candidemia in non-haematological setting

Different management strategies cen be used for managing suspected or documented IC, including prophylaxis, empirical or pre-emptive therapy and treatment of a culture-proven infection. Based on the incidence of IC, prophylaxis may be judged appropriate in patients with high risk of IC (incidence > 10%). In settings with lower incidence rate, patients might benefit from pre-emptive strategies based on predictive scores. Obviously, the knowledge of local epidemiology helps to define the most appropriate antifungal therapy, based on the most frequent species and susceptibility patterns of *Candida* isolated in a single centre.

Prophylaxis, defined as administration of an antifungal to a patient with no evidence of infection, has been evaluated in surgical and critically ill patients in several studies and metaanalyses.^33^,^64^–^73^ Fluconazole prophylaxis reduced by approximately 50% the incidence of IC, and seemed associated with improved outcome.^70^–^72^ Naturally, antifungal prophylaxis is efficacious and cost-effective in populations with high prevalence of IC, when the number of patients that need to receive the prophylactic treatment in order to prevent one episode of IC (number needed to treat) is low. On the other hand, the disadvantages of fluconazole prophylaxis include overtreatment, possible toxicity and profound influence on local epidemiology with the emergence of azole-resistant isolates.^74^ Therefore, antifungal prophylaxis might be indicated only for patients or procedures in which the rate of IC is higher than 10%, as compared to the normal rates of 1–2%.^41^,^54^,^75^ In such populations, the number needed to treat is less than 20, as compared with over 100 in an average population of ICU patients with the incidence of IC of 2%.

Empirical treatment is defined as the administration of antifungals in the presence of persistent or refractory fever in subjects who are at high risk of developing a fungal infection. This strategy has been developed almost 3 decades ago for neutropenic cancer patients, when it became evident that the lack of sensitivity of microbiological and clinical findings was likely resulting in delayed diagnosis and increased morbidity and mortality.^76^ Although the first studies on empirical therapy had numerous methodological flaws, this fever-driven strategy is being used in different clinical settings and various antifungals are recommended for empirical treatment of invasive candidiasis, both in neutropenic and non-neutropenic patients.^41^ However, in ICU or surgery patients, there are several causes of protracted fever and, probably for this reason, in a randomised multicenter study in critically ill patients, the empirical therapy with fluconazole was not more beneficial than placebo.^77^

With the availability of diagnostic tools such as radiological imaging, invasive diagnostic procedures, improved cultures techniques and serological markers, it became evident that a diagnosis-driven approach was possible and should be pursued. Pre-emptive treatment is characterised by starting antifungal therapy when one or more microbiological or clinical markers result positive. Microbiological markers include multiple colonisation, positivity of mannan, (1,3)-beta-D-glucan or molecular testing.^60^,^62^ However, there is a certain degree of confusion between prophylaxis, empirical and pre-emptive treatment in patients with high risk of IC, as defined by high *Candida* colonisation index. In fact, the IDSA guidelines recommend a pre-emptive approach (although they continue to call it empirical treatment) based on clinical assessment of risk factors, serologic markers, and/or culture data from nonsterile sites, rather than fever.^41^

Despite all the advances in diagnostic tools, it is to be remembered that repeated blood cultures, both from CVC and peripheral line, remain the cornerstone of diagnosis of candidemia, and that any positive blood culture for *Candida* must be taken seriously and needs appropriate treatment.

Broad spectrum antifungals are recommended for the first line treatment while species identification is pending, but when species is known, a de-escalation can be recommended.^41^ The initial choice of antifungals depends on patient’s clinical condition and the risk of azole-resistant strain, due to previous azole exposure or local epidemiology.^41^ For patients in severe or moderately severe clinical conditions (e.g. hemodynamically unstable, or with suspected concomitant organ involvement), echinocandins are the first choice because of their cidal activity against *Candida* and excellent toxicity profile.^41^ Liposomal amphotericin B - another fungicidal agent indicated for first line treatment in critically ill patients, is more expensive and probably associated with a higher toxicity.

## Other aspects of treating invasive candidiasis

Once the initial therapy for candidemia is started, several clinical issues remain open. First, the efficacy of the treatment should be assessed by the documentation of blood cultures returning sterile. Indeed, the date of the first negative blood culture is important, because the recommended length of treatment is 14 days after the last positive blood culture and resolution of symptoms attributable to candidemia.

Second, the antifungal chosen initially can be changed on the basis of species identification or susceptibility testing. Thus, for stable patients with *C. albicans* or other azole-susceptible strains, fluconazole probably remains the drug of choice. Fluconazole might be preferred over echinocandins for treating *C parapsilosis*, as caspofungin MICs for *C. parapsilosis* are higher than those for other *Candida* species.^41^,^78^ However, in a recent analysis of data from five clinical trials, that included 71 cases of infection due to *C. parapsilosis*, the success rate was comparable with other non-albicans species.^79^

Third, patients who improve clinically and who cleared *Candida* from the bloodstream, might be suitable for step-down oral therapy to complete the course of 14 days. The available oral antifungals are fluconazole, itraconazole, voriconazole and posaconazole. Fluconazole is an obvious choice for susceptible species, while voriconazole can be indicated as step-down therapy for *C. krusei* or voriconazole-susceptible *C. glabrata* and in ocular or cerebral infections, because of excellent tissue concentration.

Additionally, ophthalmologic fundus examination is indicated in all patients to exclude endocular infection, while endocarditis should be excluded in case of persistently positive blood cultures, known valve pathology or any other sign or symptom suggestive of endocardial involvement. As described elsewhere, in both these complicated cases the duration of treatment should be much longer (more than 4 weeks and up to lifelong suppressive therapy).^41^

Finally, intravenous catheter removal is strongly recommended for patients with candidemia. Indeed all guidelines, both on the management of candidiasis and on the management of catheter-related bloodstream infections, state clearly that catheters should be removed, even though one should admit that all statements indicate grade II or III of scientific validity of recommendation, in absence of data from properly randomised, controlled trials.^41^,^80^,^81^ However, the issue might still be controvertial since a recent study, based on a multivariate analysis of 842 adults included in candidemia trials, did not find any benefit of early central venous catheter removal (i.e. within 24 or 48 hours after initiation of antifungal therapy) on survival.^81^

## Conclusions

*Candida* is one of the most common causes of nosocomial bloodstream infection. Non-neutropenic patients now constitute a large but heterogeneous population of patients at risk of IC, which includes subjects admitted to adult or neonatal ICU, undergoing abdominal surgery and those with cancer or numerous medical comorbidities 8e.g. diabetes). Morbidity and mortality associated with candidemia are significant and the epidemiology of species have been shifting towards non-albicans strains. Even though numerous risk factors for invasive *Candida* infection have been reported and several antifungals are widely available, the optimal management of candidemia remains a challenge. Prophylaxis might be beneficial in population with incidence > 10%, while novel diagnostic techniques should be further studied to enable pre-emptive treatment in populations with lower incidence rates.

## Figures and Tables

**Table 1:** Risk factors associated with invasive candidiasis in general and candidemia due to different *Candida* species.^1^

*Candida* species	Risk factor
***Candida*in general**	Prior abdominal surgery Intravascular catheters Parenteral nutrition Use of broad-spectrum antibiotics Immunosuppression, including corticosteroid therapy Acute renal failure Diabetes Transplantation Haemodialysis Pancreatitis
***C. tropicalis***	Neutropenia and bone marrow transplantation
***C. krusei***	Fluconazole use Neutropenia and bone marrow transplantation
***C. glabrata***	Fluconazole use Surgery Vascular catheters Cancer Older age
***C. parapsilosis***	Parenteral nutrition and hyperalimentation Vascular catheters Being neonate^2^*
***C. lusitaniae*and*C. guilliermondii***	Previous polyene use
***C. rugosa***	Burns

1 Adapted from the following references: 6,31,35

2 Epidemics due to nosocomial horizontal transmission via hands of health personnel have been reported.^45^,^46^
